# Editorial: Thyroid hormones and diet

**DOI:** 10.3389/fendo.2025.1673729

**Published:** 2025-08-18

**Authors:** Joao Anselmo, Elisa Keating, Joseph V. Martin, Lutz Schomburg

**Affiliations:** ^1^ Endocrinology Department, Hospital Divino Espírito Santo, Ponta Delgada, The Azores, Portugal; ^2^ Department of Biomedicine-Biochemistry Unit, Faculty of Medicine of the University of Porto, Porto, Portugal; ^3^ Biology Department, Center for Computational and Integrative Biology, Rutgers University, Camden, NJ, United States; ^4^ Institute for Experimental Endocrinology, Charité Medical University Berlin, Berlin, Germany

**Keywords:** thyroid hormones, iodine, selenium, goiter, metabolic rate

Thyroid hormones—primarily T3 (triiodothyronine) and T4 (thyroxine)—are essential for regulating numerous physiological processes, including development, metabolism and energy production. Diet plays a crucial role in maintaining healthy thyroid function and managing thyroid disorders, especially those resulting from deficiencies or excesses in thyroid hormone levels. The purpose of this Research Topic was to collect recent updates on the role of diet on thyroid function and thyroid health.

The main reason for the diet’s importance in thyroid function is the requirement for several micronutrients—also called trace elements—that are essential for thyroid hormone synthesis but cannot be synthesized by the human body. These nutrients have to be furnished by external sources. Iodine and selenium are particularly critical. Iodine is a structural component of thyroid hormones, and its deficiency is a major cause of hypothyroidism. Selenium, in synergy with iodine, supports the activity of enzymes involved in thyroid hormone synthesis and metabolism. Other trace elements such as zinc, copper, and iron also contribute significantly to thyroid hormone production and function (1, 2). Vitamins, in particular vitamin C may also influence thyroid hormone production, as it shown by Wu et al. in a large epidemiological study.

Iodine deficiency remains a global health problem, affecting over two-thirds of the world population (3). It is especially concerning during pregnancy and early childhood due to its negative impact on brain development (4). Goiter—thyroid gland enlargement—is the most common manifestation of iodine deficiency and remains endemic in several regions, particularly in Sub-Saharan Africa. Tuke et al. reported a goiter prevalence of 37.2% among school-aged children in the Guraferda District, Southwest Ethiopia. Their findings highlight the urgent need for comprehensive iodine supplementation programs in such regions, since goiter is the alarming visible sign of iodine deficiency, whereas neuro-developmental impairments and hypothyroidism are less obvious but also of high relevance for the affected population.

Severe iodine deficiency during pregnancy can lead to profound cognitive and physical impairments in the offspring, a condition known as congenital hypothyroidism. Iodine deficiency is largely preventable through dietary supplementation, primarily via iodized salt (5). Importantly, iodine supplementation may be recommended even in coastal areas, which are believed to be abundant in natural iodine given the geographical proximity to the sea, as demonstrated by Wu et al. in a study conducted in Hainan Province, China. In fact, many coastal areas are subjected to high rainfall which can leach iodine from the soil into the sea, reducing the iodine content in local food products, including vegetables and grains, but also milk, eggs, and meat. This is notably the case in the Azores islands, where continuous iodized salt supplementation has proven effective (6).

Since iodine is not essential for plant growth, plant-derived foods typically contain low iodine levels. Consequently, individuals following plant-based diets, including vegans and vegetarians, may be at increased risk of iodine deficiency due to the absence of animal-based food sources. Croce et al. discuss these challenges in their review on iodine nutrition in vegan and vegetarian diets, emphasizing the need for education, better diagnostics and targeted supplementation strategies in these populations. This issue is even more important given the putative increased consumption of goitrogenic foods in these dietary patterns, which certainly deserves further investigation (7).

While the risks of iodine deficiency are well known, the consequences of iodine excess are less thoroughly studied (8). Diets rich in iodine, particularly those that include seaweed or excessive use of iodine-containing supplements and contrast media, can lead to systemic health issues due to the overproduction of thyroid hormones. Excessive iodine intake has also been linked to autoimmune thyroid diseases, such as Graves’ disease and Hashimoto’s thyroiditis, particularly in genetically susceptible individuals. For example, Japan, a country with a traditionally high iodine intake from seaweed, also has a higher prevalence of autoimmune thyroid conditions (9). Herein, in two different works Khudair et al. and Khudair et al. discuss several complications of iodine excess including implications in reproductive health.

Nie et al. explored the biological mechanism underlying iodine-induced goiter and identified the overactivity of the LNC89/LNC60-COL11A2 axis as a potential contributor. This pathway involves the long non-coding RNAs LNC89 and LNC60 and the collagen gene COL11A2, which showed elevated expression in thyroid tissues of mice exposed to iodine-rich diets.

There is also growing interest in how iodine nutritional status might affect the therapeutic response to radioactive iodine in patients undergoing ablative treatment for hyperthyroidism. Hu et al. demonstrated that factors such as metabolism and organ function may influence the efficacy of this therapy, with iodine levels playing a potentially modulating role.

Thyroid hormones also play a central role in modulating metabolic rate and energy expenditure (10). During caloric deprivation, such as prolonged fasting, the body adapts by converting T4 into the less active metabolite reverse T3 (rT3), and away from the biologically active T3, thereby reducing the activity of T3-dependent processes. This shift serves to reduce metabolic rate and conserve energy, an evolutionary survival mechanism that gains time by suppressing energy loss. Beyond metabolic adaptation, fasting has been associated with a range of health benefits, including enhanced autophagy, improved antioxidant capacity, and increased cellular stress resistance (11). Sui et al. review these mechanisms, emphasizing the importance of tissue-specific regulation of thyroid hormones during extended fasting periods.

It is known that thyroid hormone production follows a circadian rate (12), but the relationship between the frequency of night eating and thyroid function remains unclear. This interesting aspect was investigated by Zhang et al. showing that the frequency of food ingesting during night may alter thyroid hormone levels. This is particularly relevant in person working night shifts.

Finally, in a retrospective study conducted by Lu et al., a significant positive association was identified between *Helicobacter pylori* (HP) positivity and elevated T4 levels. Evidences suggest that dietary patterns significantly influence host vulnerability to HP infection and its subsequent clinical manifestations (13), thereby potentially affecting thyroid hormone levels.

In conclusion, Iodine, selenium, and other trace elements are essential for thyroid hormone synthesis and regulation. Daily nutrition is the key aspect to avoid deficiencies in these essential micronutrients for thyroid function and health, and often combined deficiencies prevail when the food quality is poor and the choices are few. While global efforts such as salt iodization have significantly reduced iodine deficiency disorders, challenges persist—particularly among vulnerable populations like children, pregnant women, and individuals following plant-based diets. Moreover, the health risks associated with excessive iodine intake are becoming more apparent and warrant careful monitoring. Future dietary and public health strategies must balance iodine intake to optimize thyroid health while minimizing risks associated with both deficiency and excess, and require also attention to the supply with the other essential trace elements, to provide the thyroid axis with the required substrates for full functionality and long-lasting health.
